# Why use Appreciative Inquiry? Lessons learned during COVID-19 in a UK maternity service

**DOI:** 10.18332/ejm/147444

**Published:** 2022-05-19

**Authors:** Rachel Arnold, Clare Gordon, Edwin van Teijlingen, Sue Way, Preeti Mahato

**Affiliations:** 1 Centre for Midwifery, Maternal and Perinatal Health, Bournemouth University, Bournemouth, United Kingdom; 2 Faculty of Health and Care, University of Central Lancashire, Preston, United Kingdom

**Keywords:** healthcare staff, wellbeing, appreciative inquiry, maternity, transformational change, critical reflection

## Abstract

Choosing the ‘right’ research method is always an important decision. It affects the type of study questions that can be answered. In addition, the research method will have an impact on the participants – how much of their time it takes, whether the questions seem important to them and whether there is any benefit in taking part. This is especially important when conducting research with staff in health services. This article is a reflection on the process of using Appreciative Inquiry (AI) in a study that explored staff wellbeing in a UK maternity unit. We share our key learnings to help others decide if AI will fit their research aims, as well as highlight issues in its design and conduct. We discuss our experience of using AI,the strengths and limitations of this approach, and conclude with points to consider if you are thinking about using AI. Although a study team was actively involved in decisions, this paper is largely based on reflections by the first author, the researcher conducting the field work in the maternity services.

## INTRODUCTION

Reflective practice is an essential part of professional development for midwives^1^. It is also vital that researchers reflect on how they conduct research, how their design and language choices, as well as underlying values and priorities, contribute to how data are produced^2^. Sharing these reflections is essential for the growth of the research community as well as increasing the trustworthiness of research findings. Furthermore, placing ourselves and our practices under scrutiny is an ethical imperative^3^. This article, therefore, reports the Principal Investigator’s experiences, feelings and reflections of using Appreciative Inquiry (AI) for the first time in a study on staff wellbeing in a UK maternity service.

UK maternity services were under immense pressure before COVID-19 from the combination of heavy workloads and a chronic shortage of midwives, particularly in England^4,5^. UK midwives and doctors working in obstetrics report high levels of stress, burnout and poor mental health^6,7^, particularly student midwives, early career midwives and trainee doctors in obstetrics^5,6^. It is, therefore, vital to understand how to care for and retain existing staff – to support and enhance their wellbeing.

In September 2020, in a maternity unit in the Southwest of England (seven months into the COVID-19 pandemic), we commenced an Appreciative Inquiry^8^ study that explored staff wellbeing. Our research question was: ‘What key factors support and enhance the wellbeing of maternity staff?’. Our aims were to: 1) identify the strengths of the service, 2) identify the factors that contribute to staff wellbeing, and 3) help create an environment where staff could build on the best and most meaningful aspects of their work.

Although a former midwife, the Principal Investigator was new to AI apart from background reading, an online course^9^, and discussions with AI practitioners. She, therefore, started as a novice to the AI approach. Our findings and discussions center around what she learnt about conducting an AI during the first phase of the research process.

### What is AI?

AI was first developed by Cooperrider and Srivastva^8^ and sits within action research approaches that aim to create practical and collaborative change. It has a distinctive and deliberate strengths-based approach to achieve emancipatory learning and change, rooted in the experiences of stakeholders. AI has a strong theoretical base that draws on social constructionism^10^, neuroscience^11^, and positive psychology^12,13^. Its assumptions include that: 1) every society, organization or group/team have strengths or things that work well and can be elaborated and expanded; and 2) our realities can be co-created through the quality of language, relationships, interactions and actions with one another^14^. These principles will be discussed in greater depth below.

**Figure 1:**
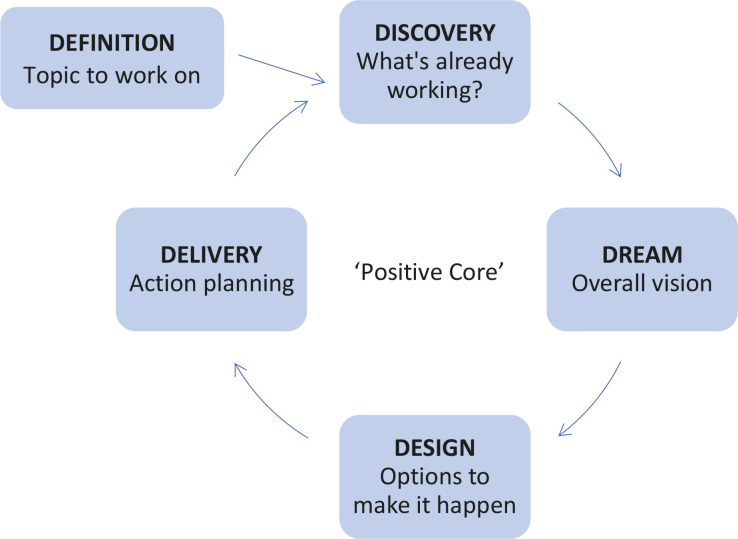
The Appreciative Inquiry change process

AI has been used in business, non-governmental organizations (NGOs), the military, coaching, organizational development, and healthcare^11,15-18^. The original model uses a four step or ‘4-D process’ (Figure 1) to take participants through an in-depth exploration of their organization, team or individual role^16^. The process starts with discovering and appreciating best experiences (discovery), imagining the ideal – how it would be if those valued experiences happened most of the time (dream), defining the dream more clearly and discussing steps towards realizing it (design), to wide ranging actions, improvisation, learning, and adjustments (destiny)^14,16^. Deciding what to study, or the ‘affirmative topic’, is important because ‘human systems move in the direction of what they study’^16^. For this reason, some have added an extra step, ‘definition’ (Figure 1)^19^.

AI is a collaborative and energizing process that enables people to connect emotionally with their situation, consider others’ perspectives and change how they see their situation, enabling them to see new possibilities and take action^20,21^. It has the potential for transformational change as people start to see compelling images of what could be – and are inspired to act^20^.

## METHODOLOGICAL APPROACH

The study was conducted in a maternity service in the South-West of England. All staff working clinically were eligible to participate. The study was advertised via staff meetings, the maternity services newsletter, notice boards, and word of mouth. Information sheets^22^ were distributed to staff who were interested in participating in an interview. To avoid any coercion, groups of staff rather than individuals were approached. Interviews were offered in-person or online. The in-person interviews were conducted in the maternity unit. Participants in online interviews were usually in their own homes.

Prior to interview, potential participants were given: 1) a brief explanation about AI, and 2) the opportunity to ask questions. Issues of confidentiality and anonymity were discussed, and informed consent was obtained^23^. The interview questions were developed following the AI process of exploring the things that are already working, imagining a future with more of these meaningful experiences and steps that can be taken towards this. Staff were asked: ‘Tell me about one of your best experiences working here’, ‘What made this such a meaningful experience?’, ‘What do you value about your work?’, and ‘What helps you to thrive and stay well despite the challenges?’. Staff were then asked to imagine how these strengths could be enhanced and built on in the future.

A broad range of staff (n=39) participated – senior and junior midwives and doctors, maternity support workers and student midwives. The interviews lasted 30–110 minutes and were digitally recorded, transcribed, and thematically analysed^24^. The themes were then checked, shared, discussed and refined collaboratively with the participants and maternity staff.

### Principles in action

The change process in AI is underpinned by five main theoretical principles^14,[Bibr cit0016]16^. Each principle is described and illustrated with reflections from our study.

*Simultaneity principle*

A key tenant of AI is the simultaneity principle which claims that change begins the moment we ask a question^16^. Questions have the potential to turn people’s attention in a different direction enabling them to see things from an alternative perspective^25^. Reliving events that have inspired us, feeling pride for challenges that have been overcome, connecting with deeply held values and the ‘positive core’ of experiences has a generative potential^11^. It builds resilience, nurtures trust, openness to new ideas, and creativity^11^. Most health and social care research generates data that can then be used to revise policies or improve practice but, with AI, the interview conversation is part of the intervention.

One midwife in our study talked about a group of women that she particularly enjoyed supporting. Probed for details, she became increasingly animated. A few days later she emailed explaining that following our interview she realized how much she cared about this area of practice. She realized that she would like to specialize in it and had approached her line-manager to discuss this. For this midwife, the interview questions had helped to initiate change because through them she had identified the part of her role that she loved the most and wanted to do more of. Inquiry and change had happened simultaneously.

*Anticipatory principle*

AI suggests that the images or mental pictures that we have of the future inspire us into actions that can make them happen^16^. If there is an image that captures the imagination and enthusiasm of an organization or team, then people will start moving towards it. One of the strengths of AI is that it is possibility-centric rather than problem-centric^26,27^. Rather than fixing a problem, AI highlights the strengths of communities and helps them to discover possibilities^26^. AI can, therefore, be emancipatory as it highlights issues of power, helps to develop critical thinking and disrupts self-limiting^21,28^.

One participant was unhappy with her work environment and colleagues. I asked what was important to her and to describe how she would like things to be. She wrote a few days later saying that she had thought about the interview a lot. She had realized that she could be part of the solution rather than a victim and had decided that she was going to do this. This participant had imagined a better future, realized that this was what she wanted, and that it was within her power to do something about it.

*The constructionist principle*

One of the tenants of AI is that ‘words create worlds’^14^, that the language and words we use not only describe reality but have the power to shape it. The stories that are told in teams, the images and language that are used mould them. Therefore, the stories that are told during AI are key, because they become part of the narrative, identity, and culture. The words and images that AI researchers use will also have a significant impact.

An interview was interrupted due to work pressures and continued the following day. The first part of the interview was filled with stories about those who inspired this staff member, the strength and joy of being a cohesive team even in the toughest of times, her aspirations, determination, and humor. Continuing the interview the next day, the participant started talking about burnout, the despair of colleagues from a previous workplace and the almost overwhelming pressures she currently felt. We eventually returned to the ‘AI space’ but the temporary switch to an antithetical state of being shocked me. After the interview, I checked the recording and noticed that when I recapped on the study rationale, I had used the words ‘staff burnout and stress’. The participant had focused on those words as she told tragic stories and started to appear overwhelmed as she listed her seemingly impossible work and study load.

Becoming a skillful AI researcher^29^ means to understand the generative power of words and to choose them with care. I wrote reflective summaries of many interviews, analyzing what worked well and noticing unhelpful words or questions so that I could develop my skills. The interview schedule became a working document – the main questions remained the same but small details and words were refined.

As part of the feedback and co-analysis many of the positive, ‘best of’, stories have been anonymized and widely shared with maternity staff. This has generated many comments, similar stories and conversations about the things that make a difference, what colleagues appreciate about each other and what helps them to do a great job. These conversations and stories are helping to shape the constantly evolving identity of the maternity services.

*The poetic principle*

The poetic principle suggests that we can choose what we study or focus on, and whatever we focus on will grow. Choosing what to study and how this will be studied is, therefore, a very important decision as it will determine what is highlighted and what will become more prevalent. In AI, the telling and retelling of stories about the things that are valued, meaningful, and represent the ‘best of’, are considered a powerful catalyst for change^14^. The decision to use AI for this study came out of this principle.

The decision to focus on staff wellbeing had been made during a period of personal orientation to the maternity services. When I shared an initial report on my orientation and study rationale with one of the senior staff, however, I was surprised by her response. She was deeply affected by two negative quotes from staff about the demands of the job – she saw this as her personal failure. She affirmed her support of the research ‘whatever was found’, but it was a stark reminder of how passionately healthcare staff care and how easy it is to inadvertently discourage. It also highlighted an internal tension between wanting to enhance staff wellbeing through finding problems that could then be ‘fixed’. This seemed incongruous. I realized that I would find ‘whatever I looked for’, that it is easier to criticize than build up, but that if I wanted to support wellbeing, I needed a different approach. I, therefore, decided to use AI, to look for the best, to amplify the strengths of the service and help the team imagine a future where they could maximize these strengths.

*The positive principle*

The positive principle claims that ‘positive questions lead to positive change’^16^. This is because positive questions shift people’s attention from problems to what gives life, what excites, energizes and nourishes them^5,16,27^. In addition, research confirms the power of positive emotions to enhance resilience, openness to new ideas and creative thinking, to building relationships and more cohesive communities^11,12^. Affirmation challenges the status quo – especially in healthcare where there is generally a deficit discourse.

As reported elsewhere^30^, being asked positive questions appeared to surprise staff. They particularly struggled when asked to talk about challenges they had overcome or things that they were proud of. Despite initial hesitation, midwives, doctors and maternity support workers shared personal stories of achievements and the determination that kept them going. They talked of deeply held values and their sense of privilege knowing they made a difference to the women in their care. One participant later commented, that, after some unsettled years, the positive questioning finally made her realize how content she was in her job.

### Reflections on conducting AI

*Is AI appropriate for staff under pressure?*

The decision to use AI had been made before the COVID-19 pandemic started. As the start date approached, therefore, we wondered if it was an appropriate methodology given the acute pressures on National Health Service (NHS) staff. A webinar series on experiences of using AI in health and social care at the beginning of the pandemic indicated that AI had the potential to encourage staff, engender pride in their work and give energy^31^. The first author remained concerned, however, whether staff would be able to engage in recalling life-enhancing experiences with the increased pressures at work and home due to the pandemic^32^.

It felt important to commence interviews by acknowledging the pandemic through inquiring how the last six months had been for participants. I wrestled with how to do this though without adversely affecting the AI interviews. An AI practitioner suggested commencing interviews with *‘before I start the appreciative interview, I would like to ask you…’*, thereby clearly delineating the ‘COVID-19 question’ from the appreciative interview. Some staff’s experience of the pandemic was summarized in a few sentences, others had experienced high levels of anxiety, frustration, anger and difficulties which they shared at length.

One participant hesitantly checked several times if she could talk about home as well as work. Once reassured, she shared the multiple pressures her family were under, at the same time as she was experiencing a crisis in her work life. The interview was deeply personal. Reflecting on this interview, it was clear that the ‘COVID question’ was important, not only because it acknowledged the tumultuous events but also because it gave space for staff to bring their whole self to the interview – not simply their professional persona. This helped to create authenticity and an open communicative space.

Asking staff *‘how has the pandemic affected you both at home and work?’* took the interview to a deeper level. This was unplanned but serendipitous. Stories of childhood dreams, pride in children, overcoming work challenges, personal and family loss, sickness, meaningful experiences, and fun events with colleagues, all merged into rich, detailed, interconnected stories of the resourceful, resilient, complex, and highly motivated people behind the uniforms.

Reservations about whether AI was appropriate in a ‘crisis situation’ were answered unequivocally by the participants. I was overwhelmed by the reaction of the midwives, doctors and maternity support workers as they told their stories, ‘relived’ events, and spoke with passion, commitment, enthusiasm and joy about their work. Many reconnected with the values that had inspired them to work or pursue a career in midwifery/obstetrics. Several staff concluded the interview by saying they felt like they had had therapy; the literature refers to the therapeutic value of qualitative studies^33^.

We concluded that AI may be an excellent tool to use in times of turmoil because it is a time when people are dealing with change, rethinking approaches and priorities in their work and private life. This may be a good time to ask questions.

*The skills of the AI facilitator*

The first challenge I encountered was that AI was much more than asking different questions, or using an unfamiliar ‘method’, it required a change in my thinking and perspective. I needed to think about my words when framing questions, probing and reflecting. When a participant talked about difficulties, my inclination would be to delve into the negative details. An AI facilitator, however, needs a new internal paradigm that enables one to explore the issue from a different angle and instigate conversations about how participants would like things to be ideally. I needed to imbibe these principles and let them affect my own way of seeing, if I was to facilitate generative transformations in others. I needed to develop a genuine curiosity about the things that were meaningful and ‘gave life’ to the participants, to probe, clarify, and ask for more details. Reflecting back became part of this journey as I learnt to notice and affirm the particular skills, courage, determination or resilience of the person sitting in front of me. In addition, as the participants recalled events in detail, I was drawn into these deeply personal often emotional stories. It was a collaborative process where both participant and facilitator were challenged, inspired and changed by the stories that were crafted together.

Conversations with other AI practitioners were an essential part of my personal journey, for practical suggestions and also for reinforcing and deepening this different way of seeing, speaking and being. There appears to be a link between the impact of AI research and the facilitator’s experience^14,18,30^. So, if this is the first time you have used AI, it is good to identify an experienced AI facilitator who can be a mentor and support your learning and development. This will increase the likelihood of achieving transformational change in the study setting.

*Interviewing using AI approach*

Interviews using AI can be time consuming. The aim is not to obtain ‘facts’ about events and experiences but it is a relational process to generate stories that are told in such detail by the participants that they connect with the emotions of the event, uncover new understandings and create purpose. It required a safe, unhurried space and often took careful probing, listening and reflecting. Some staff talked at length with little prompting – the longest interview took place on two separate occasions and was nearly two hours long. This takes significant researcher time and produces a lot of data that have to be transcribed and analyzed. It is important to consider potential lengths of interviews when designing study protocols and deciding on numbers of participants.

### Limitations and challenges of AI

*Negative experiences and AI*

One major criticism of AI is that it does not attend to negative experiences and problems^14^ like the power dynamics and abuses that can be part of complex hierarchical organizations such as hospitals. Proponents of AI are amongst those concerned that focusing on positive stories may repress ‘potentially important and meaningful conversations’^14^ and that this could be used by executives and managers to avert focus away from challenges^27^. Bushe^14^ explains that Cooperrider’s original focus was to inquire holistically into what gives life through authentic relationships rather than an exclusive focus on the positive. Embracing the negative or ‘shadow side’ of AI, is considered by others as a more holistic approach^34-36^.

Similarly, I grappled with how to conduct interviews that focused on the best and life-giving aspects of maternity services without closing down conversations about real-life challenges. Problems were acknowledged but then reframed with questions such as: ‘so in an ideal world how would you like it to be?’. In several interviews, however, it felt necessary to listen to the distress without trying to manage, reframe or control, but wait until all was said and the appreciative interview could begin or continue. This did not appear to undermine the process, but rather enabled a deeper engagement between the researcher and staff member. ‘Appreciation’ at its rawest was to appreciate the courage, commitment and resilience of the individuals who were sharing their struggles.

*Limits of AI*

AI is not a one-size-fits-all method. As with all research methodologies, AI does not claim to solve all the issues of complex hierarchical organizations or underlying power struggles. The reach of AI only extends to the power and influence of the people who take part in the process. If senior executives engage in the AI process, the potential impact is greater, and this is one reason why AI summits that include the whole workforce have been effective in large corporations. It has been heartening, however, to see that, even in our grass roots study, individuals have realized that they have the power to act and make choices, they have been encouraged, found new direction and enthusiasm, and have continued these conversations with colleagues.

## DISCUSSION

### Requirements for a successful AI study

A key factor in the ‘success’ of our study was that the Head of Maternity was enthusiastic and supported our aim to improve staff wellbeing. The support of senior managers is vital in being receptive to staff wanting to initiate changes^30^. Transformation is also more likely if there is a good social fit between the aspirations of the group and the ideals of the organisation^18^. Ideally, as many stakeholders as possible should be involved in the process and this ‘fusion of strengths’ is generally considered essential to the generative momentum of the change process^14^. Other moderators may be the organizational or group identity and whether discussion of strengths is a new phenomenon^14^.

### Strengths of AI

*Immediate benefit for staff*

Because AI is not only a research tool but also the intervention, there can be an immediate benefit for everyone who takes part. AI builds people up because it reminds them of what inspires them, keeps them going, and what they are proud of. It builds resilience, engages the creative side of their brain, and helps them to be more trusting and relational^11,20^. Sharing these often untold stories of success can make their achievements seem more real^27^. Feeling positive about their achievements can give them the confidence and energy to face other challenges. As health systems and health workers globally continue to face enormous pressures due to the pandemic, conducting research that can make a difference to staff morale is particularly worthwhile.

*Inclusive and meaningful change*

AI can help people think differently about their role, team, the way that things are done, to imagine new possibilities and be inspired to work collaboratively towards a shared vision. Rather than a management initiative, AI involves staff from all levels of the organization. The inclusive nature of AI enables all voices, including hidden ones, to be represented^27^. This process of co-creation and valuing diverse voices can result in better engagement, changes that are initiated by and meaningful to staff, and a sense of liberation and empowerment^37^. This article, which reports on the first phase of our study, describes transformation in individuals, but the second phase of the study aims to facilitate a broader transformation in maternity services. The most effective way to achieve this is for large groups of staff to hear each other’s stories, envisage the future that they want for their team and collaborate with each other to achieve their common vision^18^.

*Complements traditional health service improvement approaches*

AI is a radically different approach from usual service improvement approaches (such as the gap analyses, audits, critical incident/safety reviews) that are prevalent in health services^30^. AI does not try to fix a problem, or fill a gap in performance, but to help people connect with their inner values, the aspects of their job that give them the greatest joy. It invites staff to focus on the positive core ‘the best of’ or the things that ‘give life’^16^. Neuroscience has found that these positive emotions provide more than a transitory ‘feel good factor’, they also build confidence, enable better relationships, and improve creative problem solving^11^. So, while the traditional approaches to health service improvement are vital in maintaining standards, learning from critical incidents and improving safety, AI can make a unique contribution by tapping into the innate strengths, creativity, and enthusiasm of individual staff and teams. It provides an alternative to top-down regulation and inspires innovation and continuous learning.

## CONCLUSION

For those who are thinking about using AI, they may want to consider:

- The strengths-based approach of AI research can provide a restorative, energizing and transformative space for staff – even in the COVID-19 pandemic.
- AI is more than a research/institutional change ‘tool’ – it is a philosophical approach to research and practice development. To conduct an effective AI study the researcher will need to imbibe the principles of AI; develop personal skills such as curiosity, emotional awareness and being authentic; and develop interpersonal skills such as appreciative dialogue, coaching and nurturing, and skills in service improvement^29^.
- Ideally novice AI researchers require an experienced AI facilitator as a mentor, to offer practical guidance, support reflective practice and nurture the internal change in perspective that AI requires.
- It is important to critically reflect on the dialogue to learn what works, revise questions where necessary and develop the skills to manage the negative issues.

For AI online resources see:

https://weatherhead.case.edu/centers/fowler/business/appreciative-inquiry

https://myhomelife.uws.ac.uk/scotland/what-is-appreciativeinquiry/

https://lms.learn.sssc.uk.com/course/view.php?id=14

## Data Availability

The data supporting this research are available from the authors on reasonable request.
